# eIF4E and Interactors from Unicellular Eukaryotes

**DOI:** 10.3390/ijms21062170

**Published:** 2020-03-21

**Authors:** Daniela Ross-Kaschitza, Michael Altmann

**Affiliations:** Institut für Biochemie und Molekulare Medizin (IBMM), University of Bern, 3012 Bern, Switzerland; daniela.ross@ibmm.unibe.ch

**Keywords:** translation, eIF4E, eIF4E-interactors, yeast, protozoan parasites

## Abstract

eIF4E, the mRNA cap-binding protein, is well known as a general initiation factor allowing for mRNA-ribosome interaction and cap-dependent translation in eukaryotic cells. In this review we focus on eIF4E and its interactors in unicellular organisms such as yeasts and protozoan eukaryotes. In a first part, we describe eIF4Es from yeast species such as *Saccharomyces cerevisiae*, *Candida albicans*, and *Schizosaccharomyces pombe*. In the second part, we will address eIF4E and interactors from parasite unicellular species—trypanosomatids and marine microorganisms—dinoflagellates. We propose that different strategies have evolved during evolution to accommodate cap-dependent translation to differing requirements. These evolutive “adjustments” involve various forms of eIF4E that are not encountered in all microorganismic species. In yeasts, eIF4E interactors, particularly p20 and Eap1 are found exclusively in Saccharomycotina species such as *S. cerevisiae* and *C. albicans*. For protozoan parasites of the Trypanosomatidae family beside a unique cap4-structure located at the 5′UTR of all mRNAs, different eIF4Es and eIF4Gs are active depending on the life cycle stage of the parasite. Additionally, an eIF4E-interacting protein has been identified in *Leishmania major* which is important for switching from promastigote to amastigote stages. For dinoflagellates, little is known about the structure and function of the multiple and diverse eIF4Es that have been identified thanks to widespread sequencing in recent years.

## 1. Introduction

Unicellular eukaryotes consist of many species and have been traditionally characterized as fungi (yeasts), obligate parasites of animals such as trypasonomatids (e.g., *Leishmania* and *Trypanosoma* species) or marine prototrophs and parasites (such as those defined as dinoflagellates). It could be assumed that unicellular organisms have a simpler functional organization as compared to metazoans. The genomes of baker’s budding yeast *Saccharomyces cerevisiae* and fission yeast *Schizosaccharomyces pombe* (which were sequenced about 30 years ago) have rendered plenty of biological information to our current knowledge on eukaryotic processes. Sequencing of the genome of less well-known unicellular species has been achieved in recent years, which allows us to compare genes encoding translation factors.

In this review, we will audit current knowledge on the yeast cap-binding protein eIF4E and its interactor eIF4G as well as further eIF4E interactors. eIF4G acts as a scaffold protein for further initiation factors and forms together with eIF4E and the helicase eIF4A the eIF4F complex. More than one form of eIF4E and its interactors such as the scaffold protein eIF4G and other 4E-BPs (eIF4E-binding proteins) have been identified. In higher eukaryotes, several variants of eIF4E and eIF4G though quite conserved in their protein sequence probably fulfill specific functions. While canonical eIF4E is required for overall cap-dependent translation, other eIF4Es fulfill specific functions required under different stress conditions such as high temperature, hypoxia, or nutritional restriction. Additionally, different eIF4Es have been shown to be specifically required during fundamental developmental processes such as germ cell differentiation and embryogenesis (spermatogenesis, oogenesis) in a variety of eukaryotic model organisms such as *Xenopus laevis*, *Drosophila melanogaster*, or *Caenorhabditis elegans* [1,2,3,4,5,6,7,8,9,10,11,12].

Surprisingly, and due to the variety of ecosystems, different strategies to cope with stress and to allow for complex developmental programs of different microorganisms exist. There is no simple and uniform system describing the function and structure of eIF4Es and interactors in all eukaryotic microorganisms.

## 2. eIF4E

**Figure 1:**
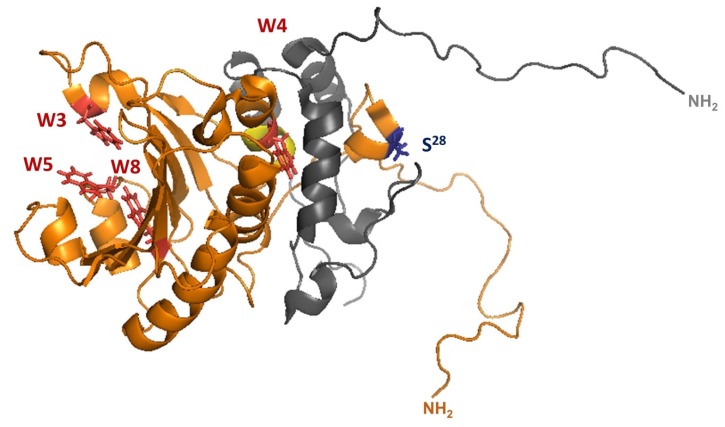
3D structure (1RF8) of *S. cerevisiae* eIF4E (gold) in complex with eIF4G (grey; aa 393 to 490). Displayed in red are aromatic residues W3, W5 and W8 in the cap-binding groove and aromatic residue W4 located at the dorsal part of eIF4E which interacts with eIF4G. Highlighted in yellow is the conserved S/TVxxF motif forming helix1 next to W4. Phosphorylated residue S28 of *S. cerevisiae* eIF4E is displayed in blue.

In 1997, the 3D-structure of both *S. cerevisiae* and murine eIF4E was solved [13,14]. Both eIF4Es share 30% sequence identity. For both, a concave part consisting of 8 antiparallel beta-sheets (β1–β8) that form the cap-binding groove is covered by three long antiparallel helices (α2, α4, and α5). Three short helical structures (α1, α3, and α6) are inserted in loops connecting beta-sheets and contribute to the convex part of eIF4E-proteins to which interactors such as eIF4G bind to. In all known eukaryotic species, eIF4E consists of a conserved core of 160-170 amino acids carrying eight conserved tryptophane residues (named W1 to W8). To illustrate these features, we present the 3D-structure of yeast eIF4E (Figure 1). The aromatic tryptophane amino acids W3 and W5 are positioned in a way that they form a stack with the ^7^m-G purine residue of the mRNA cap structure. W8 interacts in the cap-binding groove with the methyl group of the ^7^m-G residue. W4 is located at the dorsal part of eIF4E and is involved in the interaction with eIF4G or other interactors. Next to W4 is the conserved motif S/TVxxF which forms part of the dorsal helix1 interacting with eIF4G (highlighted in yellow).

Later discoveries of eIF4E variants in various eukaryotic species [7,15,16,17] opened the possibility that eIF4Es might fulfill different functions: while eIF4E class 1, which exists in all organisms, is essential for translation of capped mRNAs, further versions eIF4E class 2 and eIF4E class 3 may be required for translation of particular mRNAs or act as inhibitors of translation [15,18,19]. Not surprisingly, while most eIF4E class 1 cross-complement for *S. cerevisiae* eIF4E, eIF4E class 2 or class 

3 do not. As an example, three out of eight *Drosophila* eIF4E isoforms do not complement for the lack of *S. cerevisiae* eIF4E: eIF4E3, eIF4E6, and eIF4E8 [15,16]. While little is known about eIF4E6, eIF4E3 has been identified as a testis-specific protein required for spermatogenesis [20]. eIF4E8 or 4E-HP has been shown to act as a mRNA-specific inhibitor of eIF4E-activity during embryogenesis [15,18,19]. Variations in the arrangement of the conserved tryptophane residues has allowed for classification of metazoan eIF4Es as class 1, 2 or 3 [17,21]. While class 1 carries all eight tryptophanes, class 2 members possess W1 modified to Y, F, or L and W3 to Y or F. Rare class 3 carry W1 but W3 is replaced by C or Y. It is not clear in which way those rather conservative variations affect eIF4E activity. A clear functional assignment of these three classes is still lacking. Especially due to eIF4E’s role as a potential target in cancer treatment, much interest has been created in recent years in studying eIF4E and interactors in mammalian cells (for recent reviews, see Osborne and Borden, 2015 or Volpon et al., 2019 [22,23]).

### 2.1. eIF4E from Yeast Species

Here we describe the variety of eIF4Es and eIF4E-binding proteins from unicellular fungi subphyla Taphrinomycotina and Saccharomycotina, known popularly as yeasts. The subphylum Pezizomycotina—consisting mostly of mold species such as *Penicillium* and *Aspergillus*—is not clearly defined as uni- or multicellular and will not be further analyzed in this article.

Among the best-known and studied yeast species are *Saccharomyces cerevisiae* (budding yeast), *Pichia pastoris* (a methanogenic yeast used for overexpression of eukaryotic proteins), *Candida albicans* (used to study fungal pathogens [24]), and *Schizosaccharomyces pombe* (fission yeast). The first three species belong to the subphylum Saccharomycotina or true yeasts, the fourth to subphylum Taphrinomycotina. Though unicellular, yeast populations possess—especially when under stress—multicellular-like properties such as pseudohyphenation [25,26] and quorum sensing, increasing thereby their survival potential [27]. At the cellular level, yeast cells have developed strategies to cope with various forms of stress. Different cytoplasmic compartments called P-bodies or stress granules are preferentially formed depending on the type and the strength of stress imposed. P-bodies are mainly formed upon acute stress (such as hydrogen peroxide or other detrimental chemical treatment) and contain enzymes responsible for translation inhibition and for mRNA degradation [28,29]. Stress granules are preferentially formed upon temperature changes or nutritional deprivation (for nitrogen or glucose) and carry translationally arrested mRNA/eIF4F complexes in conjunction with other initiation factors and 40S ribosomal subunits. Upon relief of stress, both types of compartments are dissolved [30,31].

More than 30 years ago, cloning of the gene encoding for the cap-binding protein eIF4E from *S. cerevisiae* was shown to be essential for the survival of haploid cells [32] and allowed us to obtain eIF4E-mutants with the endogenous eIF4E-gene being replaced by ortholog versions of the gene from different organisms [15,33] or temperature-sensitive alleles of *S. cerevisiae* eIF4E [34,35]. This offered interesting insights into the properties of eIF4E [32,33]. Surprisingly, eIF4E from so different organisms as *Drosophila,* human or plants could complement for the essential eIF4E-activity in *S. cerevisiae* [15,33,36]. Nevertheless, not all eIF4Es are capable of substituting for yeast eIF4E, e.g., *Trypanosoma* or *Leishmania* eIF4Es do not complement in *S. cerevisiae* [37] and we will discuss why.

**Figure 2:**
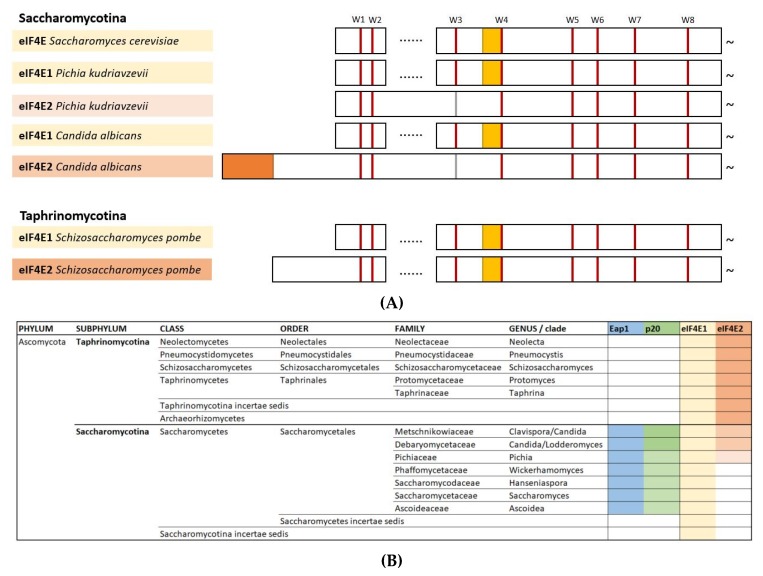
(**A**) Scheme representing the core of different yeast eIF4E1 and eIF4E2 including the eight conserved tryptophane residues W1 to W8 (labelled in red), substitutions of W3 for Y (labelled in grey). Highlighted in orange is the conserved aminoterminus (motif MSENLKRAESLFNRIMN) found in several eIF4E2s of Saccharomycotina species, marked in yellow is the helix-forming motif S/TVxxF adjacent to W4 of all shown yeast eIF4E1 and *S. pombe* eIF4E2 (but not conserved in Saccharomycotina eIF4E2s). (**B**) Phylogenetic summary of eIF4E1, eIF4E2, Eap1, and p20 from yeast species of different subphyla and families. (i) Eap1 containing species are marked in blue; (ii) p20 containing species in dark green for families Metschnikowiaceae or Debaryomycetaceae and light green for families Pichiaceae, Phaffomycetaceae, Saccharomycodaceae, Saccharomycetaceae, and Ascoideaceae. (iii) eIF4E1 (yellow) is present in all species; (iv) eIF4E2 containing species from Taphrinomycotina (dark orange), from families Metschnikowiaceae or Debaryomycetaceae (light orange) and from Pichiaceae (salmon).

While all yeasts carry (at least) one copy of eIF4E, eIF4E class 2 is found in several unicellular yeast species (see Figure 2A,B). Classification results in unicellular fungi species carrying two possible eIF4E combinations:

1.) One (sometimes two) copies of the eIF4E1 gene, e.g., *S. cerevisiae*.
2.) Besides eIF4E1, one eIF4E2 gene is thought to act as an eIF4E-inhibitor or might be required for translating specific mRNAs under stress conditions (e.g., *C. albicans* or *S. pombe*). While eIF4E1 is essential for survival, eIF4E2 is not [38].

All so far studied eIF4E1 variants from different yeast species are conserved and cross-complement *S. cerevisiae* eIF4E. We have aligned eIF4Es from subphylum Taphrinomycotina (*Schizosaccharomyces pombe*—SCHPO) and eIF4Es from Saccharomycotina species representing different families such as *Saccharomyces cerevisiae* (family Saccharomycetaceae), *Candida albicans* (family Debaryomycetaceae), and *Pichia kudriavzevii* (family Pichiaceae). Beside *S. cerevisiae* eIF4E, the other yeasts carry two distinguishable forms, eIF4E1 and eIF4E2 (see Figure 2A,B and Figure S1A,B). All eIF4E1s are similar in mass (about 25 kDa) and show a high degree of sequence conservation, especially of the eight canonical tryptophane residues W1 to W8 and surrounding amino acid sequences.

#### 2.1.1. Yeast eIF4E1 Is Phosphorylated

The aminoterminal part of *S. cerevisiae* eIF4E (aa 1-36) is unstructured and could be only determined when in a complex with the core of eIF4G (aa 393-490; Figure 1) [39]. While deletion of the aminoterminal part of yeast eIF4E (aa 1-20) does not affect its activity, further deletions diminish *S. cerevisiae* growth and past aa35 render a lethal phenotype [39].

Phosphorylation of yeast eIF4E aminoterminus has been reported before, especially of S2, S15, T22, and S28 [40]. The significance of these phosphorylation sites has remained a mystery for a long time, especially as the aminoterminus of eIF4E (aa1-20) can be erased without visible phenotypic effects [40]. Interestingly, many eIF4E1s share a serine residue at position S28 (shown in Figure 1). If not serine, this position is replaced in some yeast species by glutamic or aspartic acid mimicking constitutive phosphorylation (see Figure S1A). In a phosphopeptide analysis of different yeast species, S28 of eIF4E has been identified as an ancient conserved phosphorylation site [41]. S28 is localized at the unstructured aminoterminal part of eIF4E which interacts with eIF4G (Figure 1). Phosphorylation of S28 or phospho-mimick mutants S28D or S28E show an increased affinity for eIF4G as opposed to the non-phosphorylatable mutant S28A [41]. This renders eIF4E-phosphorylation at position S28 a good candidate for regulation of eIF4E-eIF4G interaction. Stable eIF4E/eIF4G-interaction mediated through eIF4E-phosphorylation may serve as a hallmark for arrested translation complexes being delivered to stress granules, and interaction of both factors—though essential for cap-dependent translation—may be regulated. Surprisingly, *S. cerevisiae* eIF4E S28A or S28D/S28E mutants do not show any evident phenotype (our unpublished results).

#### 2.1.2. eIF4E2 from Saccharomycotina

Not all yeast species from subphylum Saccharomycotina carry two eIF4Es, but for those that do (such as *Candida albicans* and *Pichia kudriavzevii*), a second version, eIF4E2, can be distinguished (see Figure 2A,B). Different eIF4E2s carry an extended amino- and/or carboxy-terminus and show several gaps and/or insertions when aligned with eIF4E1 (as an example, see the primary sequence of *C. albicans* eIF4E2 in Figure S1B). eIF4E2 from *P. kudriavzevii* does not show an aminoterminal extension as compared to *S. cerevisiae* eIF4E but has also several insertions and an extended carboxy-terminus (Figure 2A and Figure S1B). Additionally, W3, which is responsible with W5 to form a stack with the capped structure of the mRNA, is mostly replaced in eIF4E2s by F, Y, or other bulky hydrophobic amino acids (see Figure 2A and Figure S1B). This modification may not severely affect its cap-binding affinity as previous experiments showed that W3 (and other tryptophane residues) of *S. cerevisiae* eIF4E can be individually replaced by F without compromising its cap-binding activity and without rendering any significant in vivo phenotype [42].

*C. albicans* eIF4E2 does not cross-complement yeast eIF4E (our unpublished results). A special feature is its aminoterminal amino acid sequence which is shared among several Saccharomycotina eIF4E2s (motif MSENLKRAESLFNRIMN; highlighted in Figure 2A and Figure S1B) and which is not observed for *C. albicans* eIF4E1 that can replace *S. cerevisiae* eIF4E. Beside its extended aminoterminus and the exchange of W3 for F, *C. albicans* eIF4E2 carries several peptide insertions along its primary sequence as compared to *S. cerevisiae* eIF4E. As opposed to *S. pombe* eIF4E2, deletion of its first 56 aa does not convert it into a class 1-type eIF4E (our unpublished results). In that sense, eIF4E2 from *C. albicans* (and probably other Saccharomycotina species) differs from *S. pombe* eIF4E2. We assume that *C. albicans* eIF4E2 could act as an inhibitor of translation which becomes active under specific circumstances, e.g., nutritional or other forms of stress.

#### 2.1.3. eIF4E2 from Taphrinomycotina

Yeast species from subphylum Taphrinomycotina are distant relatives from those of the subphylum Saccharomycotina. Taphrinomycotina yeast species carry two eIF4Es which for *S. pombe* have been termed eIF4E1 and eIF4E2 [43]. While eIF4E1 is required for survival of *S. pombe*, eIF4E2 is non-essential. It is overexpressed upon salt stress under conditions when eIF4G is downregulated [38]. eIF4E2 shows reduced interaction with eIF4G and it may be required for translation of specific stress mRNAs. As opposed to *S. pombe* eIF4E1, eIF4E2 does not cross-complement for *S. cerevisiae* eIF4E [38,43,44]. Beside an extended aminoterminus, the core of *S. pombe* eIF4E2 is well conserved as compared to *S. cerevisiae* or *S. pombe* eIF4E1, especially all eight tryptophanes and surrounding sequences are present (see Figure 2A and Figure S1A,B).

We have experimentally addressed the properties of *S. pombe* eIF4E2 shortening its aminoterminus by deleting its first 28 amino acids. The shortened version of *S. pombe* eIF4E2 complements for yeast eIF4E (our unpublished results), indicating that indeed its extended aminoterminus is impeding any function equivalent to eIF4E1. Probably, the aminoterminal part of *S. pombe* eIF4E2 reduces or completely abolishes its interaction with eIF4G. Similar findings exist for variants of *Arabidopsis* eIF4E with extended aminotermini that attenuate their interaction with plant eIF4G [45]. In which way this attenuated form of eIF4E favors translation of specific mRNAs is not clear. One possibility is that mRNAs devoid of secondary structure at their 5′UTR such as those encoding for heat shock proteins and which are known to require less eIF4F for translation, are favored under circumstances when general translation is downregulated [46].

Thereby, eIF4E2s may be divided into two subgroups: those from Taphrinomycotina vs. those from Saccharomycotina and it remains unclear what the functional implications are; if eIF4E2 plays a role as either inhibiting or mitigating capped mRNA translation and thereby allowing for the preferential expression of specific mRNAs or for translational arrest under stress conditions.

## 3. Yeast eIF4E Interactors

**Figure 3:**
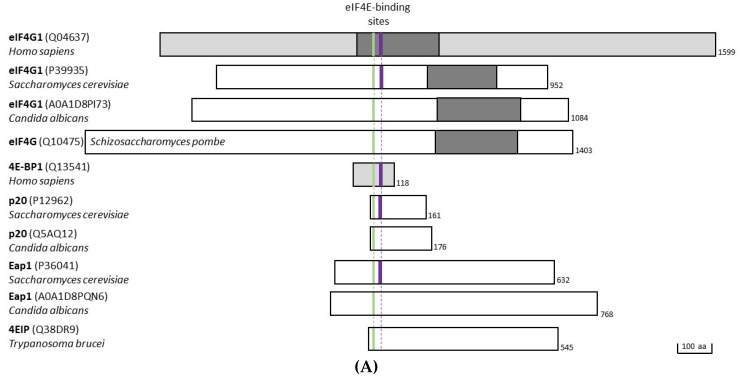

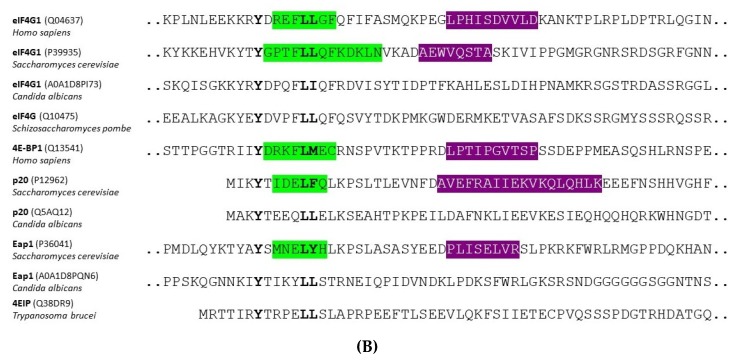
(**A**) Scheme representing eIF4G and other eIF4E-interacting proteins from different species (as indicated in the cartoon). All proteins are aligned according to their canonical (in green) and non-canonical (violet) eIF4E-binding sites that were shown directly by solving 3D structures of eIF4E in complex with the 4E-BPs (5T46, 6FC1, 4UED, 6FC3, 6FC2). HEAT/MIF-4G domains are displayed in dark grey. (**B**) Sequences of the canonical (green) and non-canonical eIF4E-binding (violet) amino acid motifs from different species aligned according to the canonical eIF4E-binding motif YxxxLΦ (bold).

Three proteins interacting with eIF4E in *S. cerevisiae* have been described: eIF4G, Eap1, and p20. eIF4G, consisting of eIF4G1/Tif4631p and eIF4G2/Tif4632p, is a scaffold protein that attracts several other initiation factors and allows for the circularization of translated mRNAs by interacting with PAB1, the poly(A)-binding protein. Eap1 and p20 are modulators that affect translation and/or stability of particular mRNAs. All eIF4E-interactors carry the canonical amino acid motif YxxxxLΦ which forms a helical structure and is required for interaction with eIF4E. As this short amino acid motif is present in many proteins (in *S. cerevisiae* around 15% of all proteins) it is not possible to predict by in silico alignments which proteins might bind to eIF4E. A characteristic arrangement of negatively- and positively-charged amino acid stretches around the canonical eIF4E-binding domain has been proposed for eIF4E-binding proteins that could electrostatically embrace capped mRNAs and thereby stabilizing mutual interactions [47]. Additional to the canonical eIF4E-binding alpha-helix found in different species (highlighted in green; Figure 3A,B), a second non-canonical motif binding to the lateral surface of eIF4E has been described in several structural studies of eIF4E/partner complexes (highlighted in violet; Figure 3A,B). This second binding motif which also forms an alpha-helix does not consist of a precise primary amino acid sequence and its distance to the canonical motif is not exactly defined [48,49,50].

### 3.1. eIF4G

eIF4G is a scaffold protein which binds several initiation factors such as eIF4E and eIF4A but also carries an interaction domain for poly(A)-binding protein and thereby allows for the circularization of translating mRNAs [51]. Especially important is its HEAT/MIF1-domain (aa 607-805 of eIF4G1 and aa 567-808 of eIF4G2 in *S. cerevisiae*), a crescent-shaped domain consisting of 10 alpha-helices arranged in 5 antiparallel HEAT-repeats [52] which is found in several proteins. It serves as an interaction platform for other proteins such as eIF4A. In higher eukaryotes, eIF4G has a second binding site for eIF4A at his carboxyterminus and also interacts with the multisubunit complex eIF3 and the MAP kinase Mnk1/2. As opposed to metazoans, yeast eIF4G is lacking the extended carboxyterminus and does not bind to eIF3 which independently, and together with eIF2, binds to 40S subunits [53]. Beside binding sites for other initiation factors, yeast eIF4G carries at least three distinct RNA-binding sites [54].

*S. cerevisiae* has two gene copies encoding for eIF4G, named TIF4631 and TIF4632. While deletion of one of the both genes is non-essential, deletion of both is lethal, indicating that eIF4G1 and eIF4G2 are functionally redundant [55]. This is reinforced by experiments where the ORFs encoding for both eIF4G1 and eIF4G2 have been interchanged in a yeast strain without causing any phenotype [56]. Studies silencing eIF4G-activity in *S. cerevisiae* indicate that it is required for effective translation of all capped mRNAs and that it is involved in recognition by 43S-preinitiation complexes of the capped 5′UTR but not for the scanning of the 5′UTR region of mRNAs [57].

Saccharomycetales species exist with only one or two genes encoding for eIF4G. While their aminoterminal part shows considerable length difference, their carboxyterminus is rather conserved both in length and in sequence (not shown). So far, no evidence for specialized functions of eIF4Gs exist in yeast. The divergence at the aminoterminus of yeast eIF4Gs does not affect the core essential part located in the HEAT-domain and we assume that it just reflects the accumulation of mutations during evolution.

Recent experiments using tagged versions of eIF4E and eIF4G suggest that interaction of eIF4E and eIF4G is dynamic [58], a notion that was already raised in the 1990s [59,60]. While actively translated mRNAs are relatively depleted for eIF4F, stabilized eIF4F-mRNA interactions are detected for translationally arrested mRNA complexes such as those found in stress granules [58]. As a simple model we propose, that eIF4E-eIF4G interaction, which is essential for cap-dependent translation [61], is disrupted during initiation and that this interaction is regulated by phosphorylation/ dephosphorylation of eIF4E (and maybe also of eIF4G). Conditions leading to the formation of stress granules would enhance eIF4E-eIF4G interaction due to phosphorylation of eIF4E and thereby block translation initiation. This could explain why translation factors associated with eIF4E/eIF4G/mRNA in stress granules (such as ASC1, STM1/TIF3, and TIF34) bind much better the eIF4E S28E than the S28A mutant [41].

### 3.2. Eap1

Eap1 is a 70 kD protein with several functions ascribed [62]. It is assumed to interact in *S. cerevisiae* with the spindle pole body. Lack of insertion of newly formed spindle pole body into the nuclear envelope leads to Eap1 specifically inhibiting translation of POM34 mRNA which encodes for a membrane protein of the nuclear pore [63]. Furthermore, a knockout of EAP1 is non-lethal but causes an increased rate of aneuploidy and a temperature-sensitive phenotype. Its function in chromosome separation during mitosis seems to be separate from its second property in competing with eIF4G for eIF4E-binding [64]. Eap1 also forms part of a regulatory mechanism whereby translation is downregulated when lipid synthesis is inhibited [65,66]. Additionally, Eap1 has been described to accelerate degradation by increasing through its interaction with eIF4E decapping of certain mRNAs [67,68]. Additionally, Eap1 has been shown to interact with the helicase Dhh1 under nitrogen-deprivation and allow by direct mRNA-binding translation of autophagy related ATG1 and ATG13 mRNAs [69].

Eap1 (as p20) only exists in Saccharomycotina (summarized in Figure 2B). Recent data suggest that Eap1 is interacting with several hundred mRNAs that are affected by Eap1′s action. The interactome of Eap1 though sharing partial identity is different from that of p20 [70,71]. Beside its eIF4E-binding domain, Eap1 does not share homology with p20 [72].

### 3.3. p20

p20 is a small, non-essential protein which is the target for multiple phosphorylation at several serine and threonine residues [40,73]. It is expressed in *S. cerevisiae* at approximately half the level of eIF4E with around 20′000 copies per cell (see also *Saccharomyces* Genome Database: www.yeastgenome.org). p20 (as Eap1) only exists in yeast species from the subphylum Saccharomycotina (see Figure 2B).

It should be emphasized that beside the conserved eIF4E-binding motif YxxxxLΦ, p20 has no homology of its primary amino acid sequence to 4E-BPs from higher organisms. Mammalian 4E-BP2 only inhibits cap-dependent translation in *S. cerevisiae* when endogenous eIF4E is replaced by human eIF4E [33,74]. So, the affinity of mammalian 4E-BPs for yeast eIF4E is much smaller than that for mammalian eIF4E [74]. p20 probably accomplishes similar functions as metazoan 4E-BPs by modulating the expression of specific mRNAs and forming a structurally similar complex with eIF4E [13]. Thus, p20/metazoan 4E-BPs represent an example of evolutionary convergence (no homology of primary amino acid sequence but similar function) [75].

Though p20 was originally assumed to be an inhibitor of translation competing with eIF4G for eIF4E, a knockout haploid ∆p20 yeast strain did not show any particular phenotype under laboratory conditions [76]. Only overexpression of p20 in yeast strains carrying mutations in eIF4E and associated proteins enhanced the phenotype of those mutations [77]. Data of the translational profile of a p20-knockout *S. cerevisiae* strain lead to the conclusion that it acts as a modulator of translation affecting preferentially cap-dependent translation of certain mRNAs [70]. Recent experiments using purified p20 in complex with eIF4E (both expressed and purified from *E. coli*) show that p20 is per se an RNA-binding protein which stabilizes a ternary complex formed by both proteins and capped mRNA [78]. Surprisingly, in vitro experiments with an eIF4E/p20-dependent yeast lysate show clear stimulation of translation when adding purified p20/eIF4E complex. The stimulatory effect is much more pronounced than when adding equimolar amounts of eIF4E alone and is observed for capped reporter mRNAs with 5′UTRs of differing length and complexity [78]. We propose that p20/eIF4E/capped mRNA form a stable complex in the cell which might be in a dynamic equilibrium with other complexes consisting of *S. cerevisiae* eIF4E and eIF4G or Eap1 and that depending on prevailing physiological conditions this equilibrium might shift towards translation initiation, mRNA degradation and/or other processes. Remodeling of distinct eIF4E-4E-BP complexes causing translational inhibition, activation or degradation of specific mRNAs as an answer to outer stimuli has been recently proposed for *C. elegans* and *Drosophila* germline and embryonic cell development [4,8].

It has been difficult in the past to associate a phenotype to the loss of p20 in *S. cerevisiae*, as we have only observed a mild temperature-sensitive phenotype (reduced growth at 37 °C). As mentioned, only yeast species from subphylum Saccharomycotina carry p20 and Eap1 (Figure 2B). We assume that p20′s (and Eap1′s) function regulating translation of particular mRNAs is specific for Saccharomycotina and/or that such a regulatory function might be accomplished by eIF4E2 in species of the Taphrinomycotina subphylum which are genetically very distant from *S. cerevisiae* (and do not have p20 or Eap1).

The aminoterminus carrying the eIF4E-binding motif and the carboxyterminus consisting of a hydrophobic motif FNAFEAL followed by several acidic residues are very conserved among all p20s [72]. Yeast species such as *S. cerevisiae* and other Saccharomycetaceae share a very conserved p20 form and carry only eIF4E1, while yeast such as *C. albicans* and related species that additionally have eIF4E2 carry a somewhat different p20 with an amino acid motif insertion in the middle part of the protein (indicated in Figure 2B). Is this correlation just reflecting phylogenetical distance? We cannot answer that question yet, but *C. albicans* p20 complements for the lack of endogenous yeast p20 in our knockout *S. cerevisiae* strains with a mild temperature-sensitive phenotype (poor growth at 37°C; unpublished results) indicates similar functional properties.

## 4. eIF4E and Interactors from Protozoan Parasites

There is a large variety of eukaryotic protozoan parasites such as *Giardia lamblia* (colonizing the small intestine and causing giardiasis) or *Plasmodium falciparum* (causing malaria). Their translation machinery has been studied intensively. They both carry eIF4E and form eIF4F complexes to promote translation of own capped mRNAs. For *Giardia lamblia*, eIF4E1 and eIF4E2 have been identified. While eIF4E2 promotes translation of ^7^mGpppG-capped mRNAs, eIF4E1 interacts with small nuclear RNAs that carry a m^2,2,7^GpppN cap-structure [79]. In the case of *Plasmodium falciparum*, beside eIF4E, eIF4G and PABP have been identified, suggesting conserved translational features in comparison with other eukaryotic species [80].

In recent years, an increasing number of publications has been dealing with properties of translation initiation factors of protozoan parasites belonging to the Trypanosomatidae family (order Kinetoplastida) such as *Trypanosoma cruzi* (causing Chagas-disease), *Trypanosoma brucei* (causing the sleepness disease transmitted by Tse-Tse flies), and *Leishmania major* (causing leishmaniosis that particularly affects macrophages and dendritic cells). Trypanosomatidae have a digenetic life cycle (two or more hosts through the course of their life cycle), e.g., *Leishmania* parasites cycle between sand-fly vectors and mammalian hosts. In the salivary gland of the fly, the promastigote corresponding to the infective flagellated form predominates. After infection through the bloodstream of host mammalian cells such as macrophages, the non-flagellated amastigote becomes dominant. Conversion from promastigote to amastigote involves several differentiation stages and a reprogramming of the protozoan translatome.

**Figure 4:**
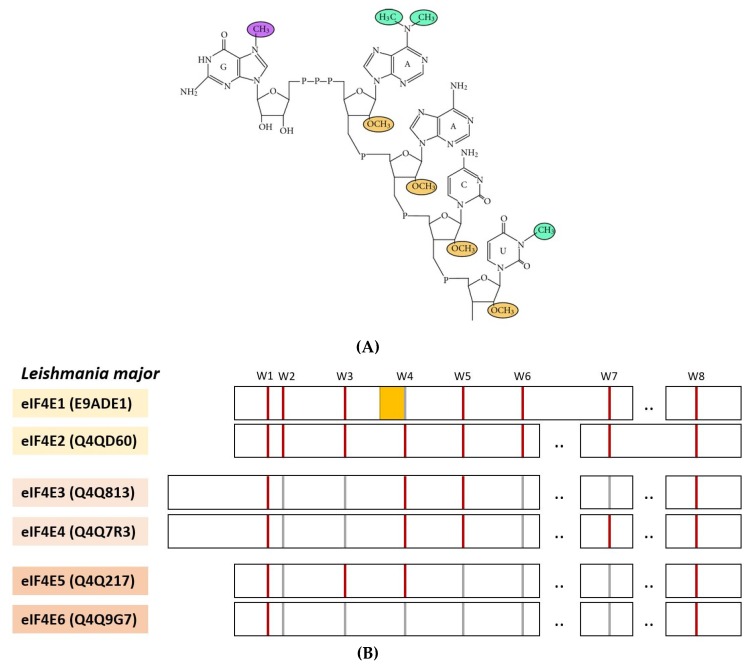
(**A**) Chemical representation of cap4-structure from Trypanosomatids (taken from Zinoviev and Shapira [81]). (**B**) Scheme representing the core of *Leishmania major* eIF4E1 to eIF4E6 (accession numbers in brackets) including the eight conserved tryptophane residues W1 to W8 (highlighted in red), substitutions of W2, W3, W4, W5, W6, or W7 for Y, F, or L are labelled in grey. Highlighted in yellow is the conserved helix-forming motif S/TVxxF next to W4 of *Leishmania* eIF4E1.

One particular feature of protozoan mRNAs is the unusual 5′ structure as the result of primary polycistronic transcript processing to form monocistronic polyadenylated mRNAs with a trans-spliced 39 nucleotide sequence at the 5′UTR carrying a cap4 structure. The unusual 5′ structure consists of m^7^Gppp_3_^6,6,2^Apm^2′^Apm^2′^Cpm_2_^3,2′^U which means that amino group N6 of adenine residue 1 is bimethylated, the imino group (at position 3) of uracil residue 4 and 2′-OH ribose moiety of residues 1 to 4 are methylated (see Figure 4A) [81]. mRNA trimethylation of residue at position 1 is also observed for other unicellular protozoans such as dinoflagellates (see below) and for certain higher eukaryotes such as *C. elegans*.

Very recently, a first crystal structure of *Trypanosoma cruzi* eIF4E5 in complex with cap4 has been obtained [82]. The interaction of *T. cruzi* eIF4E5 with cap4 shows notable differences to previously obtained ^7^mGpppG-eIF4E-structures due to the hydrophobic contacts of the additional methyl groups at 2′-OH ribose residues 1 to 4 and at adenine 1 and uracil 4 residues of the cap4 structure [82].

**Figure 5:**
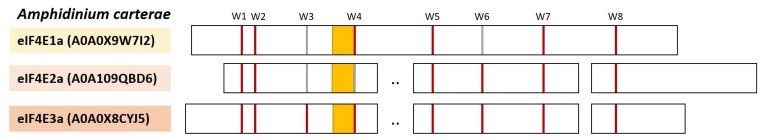
Scheme representing the core of dinoflagellate eIF4E1a, eIF4E2a, and eIF4E3a from *Amphidinium carterae* (accession numbers in brackets) including the eight conserved tryptophane residues W1 to W8 (labelled in red), and substitutions of W3, W4, and W6 for Y or F (labelled in grey). Highlighted in yellow is also the conserved helix-forming motif STVxxF next to W4.

At least 6 different eIF4E (see Figure 4B) and 5 eIF4G paralog genes have been identified for trypanosomatids (properties summarized in Table 1). For a recent review on protozoan parasites, see Freire et al., 2017 [83]. Certain protozoan eIF4E/eIF4G combinations may be required during different stages of the complex life cycle of these parasites. eIF4E1 and eIF4E2 are less abundant and do not form eIF4F-like complexes. eIF4E3 and eIF4E4 are considered as the canonical translation factors required for cap4-dependent translation. They are more abundant and carry an aminoterminal extension not observed for other eIF4Es (see Figure 4B). *Leishmania* eIF4E4 is essential for growth of the parasite [84], deletion of a single eIF4E3 allele (out of two) results in altered morphology and impaired infectivity of the parasite [85]. While *Leishmania* eIF4E3 interacts with eIF4G4, eIF4G3 interacts with eIF4E4, but not with eIF4E1, eIF4E2, or eIF4E3 [86,87]. eIF4E5 and eIF4E6 are rather small, their core eIF4E-structure is less conserved, and they are not supposed to be involved in general translation [88]. *Trypanosoma* eIF4E5 interacts with eIF4G1 and eIF4G2. Knockdown of eIF4E5 results in the loss of motility, so its activity is related to provide flagellar movement to the parasite [88]. *Trypanosoma* eIF4E6 interacts with eIF4G5 and forms a cytosolic complex with G5-IP, an eIF4G5-interacting protein. eIF4E6 knockdown causes flagellum loss [89].

None of four tested *Leishmania* eIF4E genes 1-4 complements for *S.cerevisiae* eIF4E [37], even though at least *Leishmania* eIF4E1 is able to bind to the conventional ^7^mGpppG cap structure [96,97] and *Leishmania* eIF4E2 contains the eight canonical tryptophane residues typical for class 1 eIF4E (see Figure 4B). For eIF4E3 to eIF4E6, several canonical tryptophanes are replaced by phenylalanine, tyrosin, or even other hydrophobic amino acids such as isoleucine, leucine, or valine (see Figure S2). This does not impede their interaction with the cap4 structure as shown for *Trypanosoma* eIF4E5 (see above; [82]). The only tryptophane residues that are absolutely conserved among *Leishmania* eIF4Es are W1 and W8 (Figure 4B). Not unexpectedly, trypanosomatid eIF4Es have structurally evolved differently as compared to other species due to its interaction with the unusual cap4 structure.

Interestingly, *Leishmania* eIF4E4 is capable of binding directly to PAB1 via 3 conserved motifs located in its aminoterminal domain, forming thereby beside the well-known eIF4E-eIF4G-PAB1 bridge an unusual cap4-eIF4E-PAB1-polyA bridge to circularize mRNAs involved in translation [84,95]. eIF4E4-PAB1 interaction leads to multiple phosphorylation of the extended eIF4E aminoterminus which is essential for the activity of the protein. So, beside binding to eIF4G3, eIF4E4 binds directly to PAB1 and this interaction is essential for translation [84].

Initially, *Leishmania* eIF4E4 was shown to form an eIF4F complex with eIF4G3 via a canonical YPGFSLDE motif [87]. Later studies indicate that eIF4E3 (which has only a weak cap-binding activity) interacts through a non-canonical alpha-helix of eIF4G4. Upon nutritional stress, this interaction ceases and eIF4E3 enters stress granules [85,94].

*Leishmania* eIF4E1 has been studied intensively in recent years. While eIF4E4 predominates in promastigotes (the flagellated and elongated infective form of *Leishmania*), in amastigotes (the non-flagellated form replicating in infected cells) eIF4E4 decreases and is replaced by eIF4E1 which does not interact with eIF4G [90]. An eIF4E1-knockdown is viable but the parasite cannot form the infective flagellated promastigote form [85]. In vitro, *Leishmania* eIF4E1 forms a complex with the carboxyterminus of eIF3a in the absence of eIF4G3 [92]. *Leishmania* eIF3 consists of twelve subunits and interacts with further initiation factors such as eIF1, eIF2, and eIF5 and eIF4G establishing a link between the eIF4F-complex and the 40S ribosomal subunit. It is assumed that the eIF4E1-eIF3a complex can stimulate translation of stage-specific mRNAs [83].

In promastigotes, but not in amastigotes, eIF4E1 interacts with 4E-IP (eIF4E-interacting protein) through a canonical YTREELL motif. Additionally, 4E-IP which is constitutively expressed, only interacts with eIF4E1 during the promastigote stage [90]. It has been proposed that 4E-IP allosterically destabilizes interaction of *Leishmania* eIF4E1 with the cap4 structure [91]. Bloodstream forms of the parasite lacking 4E-IP are defective in translational suppression and cannot develop into the procyclic form which initiates the promastigote life cycle [98].

In *Trypanosoma brucei* eIF4E2, the less well known eIF4E paralog which does not interact with eIF4G has been shown to interact with the 3′UTR-located histone mRNA stem-loop-binding protein SLBP2. Both proteins interact directly through a central protein segment which is missing in other SLBPs and does not carry the canonical eIF4E-binding motif [93]. A precedent for active 5′-3′ interaction during translation was previously established in *Xenopus* and mammalian oocytes. Histone mRNAs carry a 3′UTR stem-loop structure to which SLBP binds. Vertebrate SLBP interacts through an unknown factor X with eIF4E rendering histone translation cap-dependent [99,100]. In that sense, SLBP acts as a molecular mimic of PABP to circularize 5′-3′ ends of histone mRNAs during translation.

In summary, the variety of protozoan eIF4Es and interactors is much larger than that of other unicellular organisms, such as yeasts. This is probably due to their complex life cycle involving several differentiation stages. The diverse repertoire is extended by translational repressors such as 4E-IP which is used by the parasite to allow for reprogramming of protein expression when facing varying conditions in different hosts [98].

## 5. eIF4Es from Dinoflagellates

According to phylogenetic studies, dinoflagellates belong together with apicomplexans and ciliates to a huge monophyletic group called alveolates. Dinoflagellates are eukaryotic microorganisms that exist in a variety of ecosystems under different natural conditions. They can subsist as predators, photoautotrophs, or intracellular parasites. Though some dinoflagellates are parasites, most are either free-living predators or phototrophs in aquatic habitats [101]. Many photosynthetic dinoflagellates are also consumers of bacteria and other microeukaryotes. Dinoflagellate species are very important players in oceanic nutrient cycles, and some cause harmful algal blooms when cell densities reach exceedingly high levels [101]. Dinoflagellate species vary enormously considering the size of their genome which ranges from a few to a hundred thousand Mbytes and they have particular nuclear features (e.g., permanently condensed chromosomes) that make them very different from other eukaryotes. Similar to protozoan parasites, their mRNAs are also trans-spliced from polycistronic primary transcripts to form cap4-like monocistronic mRNAs and several options have been identified as alternative for the nucleotide that follows the first trimethylated base [21]. As for protozoan parasites described in this review (Trypanosomatidae), regulation of gene expression is posttranscriptional [102].

The huge differences in DNA content among dinoflagellate species is due to several genome duplications and results in species with several eIF4E gene copies (up to 15 or more). Phylogenetical studies have led to the classification of nine core dinoflagellate sub-clades organized in three different eIF4E-groups (class 1 consisting of sub-clades 1a to 1d, class 2 consisting of sub-clades 2a and 2b, and class 3 consisting of sub-clades 3a to 3c) [21]. As for other organisms, all dinoflagellate eIF4Es consist of a core amino acid sequence of around 120 amino acids with the characteristic eight tryptophane residues. As an example, we present an alignment of eIF4E of sub-clade 1a, sub-clade 2a, and sub-clade 3a from the dinoflagellate *Amphidinium carterae* (see Figure 5 and Figure S3). For class 1, W3 is replaced by Y and W6 for F, for class 2, W3 and W4 are replaced for Y. Class 3 carries all eight tryptophane residues [6]. All three classes carry the conserved S/TVxxF motif next to W4/F4 (see Figure 5 and Figure S3). Though sub-clade 3a of dinoflagellate species *Amphidium carterae* looks like class 1-eIF4E (W1 to W8 conserved), it is difficult to predict which class fulfills which function (general vs. specialized eIF4E) as no functional or structural studies of dinoflagellates eIF4Es have been published.

## 6. Summary and Conclusions

Yeast species such as *S. pombe* or *C. albicans* carry more than one eIF4E gene copy. eIF4E2 was probably “invented” in yeast species to allow for translation of specific mRNAs or to inhibit general capped mRNA translation under a variety of stress conditions. Simple eIF4E2s, such as the one from *S. pombe*, show an attenuated eIF4G interaction which was probably introduced during evolution by addition of extended aminotermini. Complex eIF4E2s, such as the one from *C. albicans* diverged from an original eIF4E1 to accomplish other functions, possibly repression of translation for a reprogramming of proteins expressed to accommodate to environmental changes. It is known that *C. albicans* pathogenicity depends on its switch from a unicellular to a multicellular hyphal form that helps to forage the environment for nutrients and probably also to adapt to changing environmental conditions.

The repertoire for potential regulation of translation was presumably augmented with the acquisition of eIF4E-interactors such as p20 and Eap1 which only exist in Saccharomycotina (for a summary, see Figure 2) and affect the translation of a subset of mRNAs by mechanisms which are not yet well understood [71]. It is clear that neither p20 nor Eap1 are inhibitors of cap-dependent translation but that their interaction with eIF4E can even favor the translation of certain subsets of mRNAs. It is surprising that a knockout of p20 in *S. cerevisiae* does not produce a strong phenotype. A possible explanation is that multiple affecting parameters are encountered by yeast cells in free nature (such as simultaneous changes of humidity, temperature, nutrition) that are not simple to mimic under laboratory conditions. It is also possible that yeasts have developed several parallel pathways to cope with different stress conditions.

More complex is the scenario for protozoan parasites such as *Leishmania* and *Trypanosoma* where several paralogs of eIF4E and eIF4G exist. This is due to different life stages requiring particular reprogramming of expressed proteins. As mentioned, different variants of eIF4E in conjunction with partner 4E-BPs also help to regulate mRNA selection under differing developmental conditions in multicellular eukaryotic organisms [1,2,3,4,5,6,7,8,9,10,11,12]. As mainly post-transcriptional regulation of gene expression exists for protozoans, it may require variations of initiation factors to favor expression of certain stage-specific mRNAs. The details of reprogramming at the level of translation initiation are not yet well understood. At least two different types of eIF4Es can be distinguished: general cap4-dependent eIF4Es (such as eIF4E3 and eIF4E4) and specialized eIF4Es (such as eIF4E1, eIF4E2, eIF4E5, and eIF4E6) which are required to allow for the synthesis of specific proteins. Additionally, inhibitors of eIF4E-cap4 interaction such as 4E-IP may be required when translation is downregulated during the passage from the amastigote to the premastigote stage.

Little is known about eIF4E and their interactors in alveolates such as marine dinoflagellates. We predict that eIF4Es and interactors from this large and heterogeneous group of eukaryotic microorganisms will become an exciting field of investigation in coming years.

## Figures and Tables

**Table 1:** Summary of reported Trypanosomatid eIF4E-interactions from *Leishmania major* (Lm) and *Trypanosoma brucei* (Tb).

eIF4E Source	eIF4E-Binding Protein	eIF4E-Binding Motif	Function/Significance	Reference
LmeIF4E1	Lm4E-IP	7-YTREELL-13	Inhibitory complex	[90,91]
LmeIF4E1	LmeIF3a		eIF4G3 not involved	[83,92]
TbeIF4E2	TbSLBP-2 (binds to stem-loop motif in 3′UTR of histone mRNA)	Central part of TbSLBP-2 which does not carry the canonical 4E-binding motif	Translation of histone mRNA	[93]
LmeIF4E3	LmeIF4G4	22-LADILAFRDT-31	eIF4F complex, dissociates upon heat stress	[94]
LmeIF4E4/(LmeIF4E1)	LmeIF4G3	20-YPGFSLDE- 27	eIF4F complex (LmeIF4A1 also detected)	[87]
LmeIF4E4	LmPAB1		5′-3′-mRNA circularization	[84,95]
TbeIF4E5	TbeIF4G1 TbeIF4G2			[88]
TbeIF4E6	TbeIF4G5 (TbG5-IP)			[89]

## Supplementary Materials

Supplementary materials can be found at https://www.mdpi.com/1422-0067/21/6/2170/s1.
