# Supplementary material for: eIF4E and Interactors from Unicellular Eukaryotes
Source: Int J Mol Sci. 2020 Mar 21;21(6):2170. doi: 10.3390/ijms21062170 (PMC7139794; doi:10.3390/ijms21062170)
Supplement: Supplementary file 1 [file ijms-21-02170-s001.pdf]

Supplementary Materials

```
C4Y5P9|C4Y5P9_CLAL4         ------------------MS-EDLTKKTE------ELSLDSEKTVLSSKEEFTAKHPLNS 35
Q9P975|IF4E_CANAL           ------------------MS-EELAQKTE------ELSLDS-KTVFDSKEEFNAKHPLNS 34
Q6BXX3|Q6BXX3_DEBHA         --MKVF------TNKIAKMS-EELSKQTE------ELSLENKDTVLSNKEEFTAKHPLNN 45
C5DJV3|C5DJV3_LACTC         ------------------MSVEEVTQKTG-D-----LNIDEKSTVLSSEKEFQLKHPLNT 36
P07260|IF4E_YEAST           ------------------MSVEEVSKKFE-ENVSVDDTTATPKTVLSDSAHFDVKHPLNT 41
I2JS39|I2JS39_DEKBR         MQMNLVGRTFPASERTRQSREEKPVE----AEVAKPEEEKKDVTVLENKEEFTVKHPLNS 56
A0A099P1Q5|A0A099P1Q5_PICKU ------------------MSTEELNN----ATKDLSLDEKKDVTALENPAEFNVKHPLNS 38
P78954|IF4E1_SCHPO          ------------------MQTEQPPKESQTENTVSEPQEKALRTVFDDKINFNLKHPLAR 42
                                                 *.  :                 *.:..  .*  ****

C4Y5P9|C4Y5P9_CLAL4         KWTLWYTKPQTNKSETWSDLLKPVITFSSVEEFWGIYNSIPVANQLPMKSDYHLFKEGIK 95
Q9P975|IF4E_CANAL           RWTLWYTKPQTNKSENWHDLLKPVITFSSVEEFWGIYNSIPPANQLPLKSDYHLFKEGIR 94
Q6BXX3|Q6BXX3_DEBHA         KWTLWYTKPQVNKSENWHDLLKPVITFSSVEEFWGIYNSIPQANQLPMKSDYHLFKEGIK 105
C5DJV3|C5DJV3_LACTC         KWTLWYTKPPVDKSESWSDLLRPVTSFETVEEFWAIHNAIPKPRYLPLKSDYHLFRNDIR 96
P07260|IF4E_YEAST           KWTLWYTKPAVDKSESWSDLLRPVTSFQTVEEFWAIIQNIPEPHELPLKSDYHVFRNDVR 101
I2JS39|I2JS39_DEKBR         KWTLWYTKPAVDKNESWADLLKPIVSFDTVEEFWGIYHAVPKAVDLPLKSDYHLFRNDIK 116
A0A099P1Q5|A0A099P1Q5_PICKU TWTLWYTKPAVDNTESWADLLKPVVTFNTVEEFWGIFHAIPKVNELPLKSDYHLFRGDIK 98
P78954|IF4E1_SCHPO          PWTLWFLMPPTPG-LEWNELQKNIITFNSVEEFWGIHNNINPASSLPIKSDYSFFREGVR 101
                             ****:  * .     * :* : : :*.:*****.* : :     **:**** .*: .::

C4Y5P9|C4Y5P9_CLAL4         PEWEDEQNAKGGRWQYSFNNKRDVAQVINDLWLRGLLAVIGETIEDD---ENEVNGIVLN 152
Q9P975|IF4E_CANAL           PEWEDEANSKGGKWQFSFNKKSEVNPIINDLWLRGLLAVIGETIEDE---ENEVNGIVLN 151
Q6BXX3|Q6BXX3_DEBHA         PEWEDEQNSKGGKWQFAFNNRRDVGSIINDLWLRGLLSVIGETIEDD---EDEVNGIVLN 162
C5DJV3|C5DJV3_LACTC         PEWEDPTNAQGGKWTHQFWGKT---PDIDDLWMRALLAVIGETIDED---DSEINGVVIN 150
P07260|IF4E_YEAST           PEWEDEANAKGGKWSFQLRGKG---ADIDELWLRTLLAVIGETIDED---DSQINGVVLS 155
I2JS39|I2JS39_DEKBR         PEWEDXANAEGGKWSHQFRSKN---IDIHEIWTRALLSVIGETIEDD--DQTEVNGVVLN 171
A0A099P1Q5|A0A099P1Q5_PICKU PEWEDSQNSDGGKWFCQFKGKR---EDMNELWTRTLLSVIGETIEKAETETNEVNGVVFN 155
P78954|IF4E1_SCHPO          PEWEDVHNKTGGKWAFQNKGRG--GNALDEMWLTTVLAAIGETLDPT---GQEVMGVVIN 156
                            *****  *  **:*      :      :.::*   :*:.****::       :: *:*:.

C4Y5P9|C4Y5P9_CLAL4         IRRANIRVGIWTKDCD-ESKLVTIGERFKKVLKLGDDQKVEFMSHDTFNAKGNAQITV-- 209
Q9P975|IF4E_CANAL           IRKQAYRVGIWTKDCD-ESKLKTVGERLKKVLQLNDEQKVEFMSHDASNTRGAEPQIVL- 209
Q6BXX3|Q6BXX3_DEBHA         IRKQVIRIGLWTKDCD-ETKLRTVGERLKKILKLSEEQKLDFMSHEDSGKKGAEPLISL- 220
C5DJV3|C5DJV3_LACTC         IRKSAYKIAIWTKSADNKEALTRIGSKFKTVLKLGDDAQLEFLPHSTASEKHPQPSLVL- 209
P07260|IF4E_YEAST           IRKGGNKFALWTKSED-KEPLLRIGGKFKQVLKLTDDGHLEFFPHSSANGRHPQPSITL- 213
I2JS39|I2JS39_DEKBR         VRRNGFKIGLWTKSCD-IEKLKPIGTRFKKVLKLSDRDTIEFMKHNNYGDRNAAPLITM- 229
A0A099P1Q5|A0A099P1Q5_PICKU VRRGTCKIGLWTKSCD-EERLRAIGEVFKKVLKLGDEDKIEFIRHKDSDNRNAKPMIIM- 213
P78954|IF4E1_SCHPO          MRKGFYRLAVWTKSCNNREVLMEIGTRFKQVLNLPRSETIEFSAHEDSSKSGSTRAKTRM 216
                            :*:   :..:***. :    *  :*  :* :*:*     ::*  *.  .

C4Y5P9|C4Y5P9_CLAL4         --
Q9P975|IF4E_CANAL           --
Q6BXX3|Q6BXX3_DEBHA         --
C5DJV3|C5DJV3_LACTC         --
P07260|IF4E_YEAST           --
I2JS39|I2JS39_DEKBR         --
A0A099P1Q5|A0A099P1Q5_PICKU --
P78954|IF4E1_SCHPO          SV 218
```

(A)

```
P07260|IF4E_YEAST           ------------------------------------------------------------
Q6BHB4|Q6BHB4_DEBHA         MSENLKRAESLFNRIMNQNGMSGRTDSNSSSSKNVANPNYNQSNNTSHNNSNGHNASGQN 60
A0A1D8PNX8|A0A1D8PNX8_CANAL MSENLKRAESLFNRIMNQTTNNDSSTSSFASQSSTPTTPISESSPTTSNTTTA------- 53
C4Y0X7|C4Y0X7_CLAL4         MSENLKRAESLFNRIMNPNKPQQETRKTSN------SNNYRQNNGSSHTHSNGS------ 48
O74743|IF4E2_SCHPO          ------------------------------------------------------------
A0A099P4P5|A0A099P4P5_PICKU ------------------------------------------------------------

P07260|IF4E_YEAST           -----MS------------------VE--EVSKKFEENVSVDDTTATP------KTVLS 28
Q6BHB4|Q6BHB4_DEBHA         NHSQSQNNHHSHSHHKTYKYNKASNY--NNNFNGQFNQPANIDLPKRDPLK----LSQET 114
A0A1D8PNX8|A0A1D8PNX8_CANAL NTSTSRSNIL-----GSRKFNSS----------SYSVPTVSSNIPKLDIEK----LTREA 94
C4Y0X7|C4Y0X7_CLAL4         ------NN---SHHRHSYKYNRYNHT-------NTPAASVVADLPKRNPAQ----EAADA 88
O74743|IF4E2_SCHPO          -----MADAEDSRHSKNEGFPNTSLITEKLDLLDLFGSPKVKTEREGRPARLLEGLSAVN 55
A0A099P4P5|A0A099P4P5_PICKU --------------------------------------------------MSKIFDNIT 9

P07260|IF4E_YEAST           DSAHFDVKHPLNTKWTLWYTKPAVDKSE-------------------------------- 56
Q6BHB4|Q6BHB4_DEBHA         LDDIPENHHVLPYCWTIWHHSRSRPKQKELININPEPVSEDAN--SEDSGQQAAAAVDSY 172
A0A1D8PNX8|A0A1D8PNX8_CANAL LASVPESHHTLPYCWTIWYHSRNKKKEQGQKVSEELQESIEEQ--SPEQQQQQSVGVDSY 152
C4Y0X7|C4Y0X7_CLAL4         VALLSSSEHVLPYCWAIWHHSRTKRVAEENN----------DD--ASSNNSTKTKGADSY 136
O74743|IF4E2_SCHPO          AETAFVKTHPLQHEWTLWFLKPPTQ-GL-------------------------------- 82
A0A099P4P5|A0A099P4P5_PICKU TASKHEETHTLSYEWNFWHHLRKPKTSSANPDSGNINTNSGTNSIPKLDDENCHRPLERY 69
                                    * *   * :*.

P07260|IF4E_YEAST           -------S---W-SDLLRPVTSFQTVEEFWAIIQNIPEPHELPLKSDYHVFRNDVRPEWE 105
Q6BHB4|Q6BHB4_DEBHA         LQTTNEIEFSAV-HDQKASTKNIGSLEQLWLSMSSLKKTYELAIGTELLIFKSGINPVWE 231
A0A1D8PNX8|A0A1D8PNX8_CANAL LQTTNELEFIDF-STG-NPIHHIASLEQLWSSFSSIKKTYTLNIGTEFLIFKTGVNPVWE 210
C4Y0X7|C4Y0X7_CLAL4         LQNTTEIEFPQY-GHPDRKIKTIASLEQMWLSLSTLKKSYNLAYGTELLVFKAGVNPVWE 195
O74743|IF4E2_SCHPO          -------E---W-SDLLKEIISFKTVEEFWGIFKTISKASMLPAKSDYSYFLKGIRPEWE 131
A0A099P4P5|A0A099P4P5_PICKU LQGMSLMEFPQIYGQGAEKTSKIDTIEQYWEALVNLKDISKLEIDTELYLFKKDIKPLWE 129
                                   .              : ::*: *  : .: .   *   ::   *  .:.* **

P07260|IF4E_YEAST           DEANAKGGKWSFQLRGKGAD-------------------IDELWLRTLLAVIGETIDEDD 146
Q6BHB4|Q6BHB4_DEBHA         DPMNTKGGRWVFRFNRRSNTGSNASNINDNDTVSKVRQRTSLIWERLLIKTITGSIIPEG 291
A0A1D8PNX8|A0A1D8PNX8_CANAL DPINSKGGRWVFRFNRKIMDGYNNNDKK-SNKQELFRKRTSLIWERLVLKTLTGSLIPDS 269
C4Y0X7|C4Y0X7_CLAL4         DPNNAKGGRWVFRFNHRNNSNSLSDDY--QETVRAGRRRATLIWERLLLKTLAGSIIPEQ 253
O74743|IF4E2_SCHPO          DPQNMNGGKWAYQSKHKGSN-------------------LDELWLYMVLAAIGETLDPTG 172
A0A099P4P5|A0A099P4P5_PICKU DEKNLNGGRWVACFGLKNFADN--------------SAWLSSLWELLSLKLISGQFISTE 175
                            *  * :**:*      :                         :*    :  :   :

P07260|IF4E_YEAST           S----------------------------------QINGVVLSIRKGGNKFALWTKSE-D 171
Q6BHB4|Q6BHB4_DEBHA         N-----------------------YSEEIQELLLNDICGLVLSVRKDEDIISIWNCNLNF 328
A0A1D8PNX8|A0A1D8PNX8_CANAL N-----------------------S-SEIQELLLNDICGLVLSVRKDEDIISLWNSNLNF 305
C4Y0X7|C4Y0X7_CLAL4         A-----------------------QN--KVSQMLGDIVGLVLSVRRDEEIISVWNSNLHF 288
O74743|IF4E2_SCHPO          K----------------------------------EVTGVVCNMRKGFYRIAVWTRNCND 198
A0A099P4P5|A0A099P4P5_PICKU ISLPLSEKLIDNPAFDNCKSSKVMSNEELNKMVIEDIAGLAVSVRNKKILISIWNTNLAY 235
                                                               :: *:. .:*.    :::*. .

P07260|IF4E_YEAST           KEPLLR-------------------------------IGGKFKQ-------VLKLTD--- 190
Q6BHB4|Q6BHB4_DEBHA         NKKRVNPEEKD------------------EDRKNQTKKLTSFQARRIICDSILRVIRECD 370
A0A1D8PNX8|A0A1D8PNX8_CANAL FGK----------------------------KDQTKKLTSFQARRIICDSILRVIRECD 336
C4Y0X7|C4Y0X7_CLAL4         GRKTG-------------------------DDDDDRKKLTPFQARRVICDAVLRVIRECD 323
O74743|IF4E2_SCHPO          KDVLEK-------------------------------IGLRFKE-------VLGISD--- 217
A0A099P4P5|A0A099P4P5_PICKU EEYKKLNGIIETVKSEEYMSYFKEPVYTGNRSNYEKCGLTRILFRKLIYQTIVDTMDEVT 295
                                                                     :         ::

P07260|IF4E_YEAST           ---------------------DGHLEFFPHSSANGRHPQ--------PS----------- 210
Q6BHB4|Q6BHB4_DEBHA         LISQGSDCISTLDSGSNERVFGVSFEYRLHSDNNNPS---IINNGGEGK----------- 416
A0A1D8PNX8|A0A1D8PNX8_CANAL LINQGSDCVETIDTGSNERVFGVSFDYRIHADNEREREKSIASNGANGSTTGGSTGSNSG 396
C4Y0X7|C4Y0X7_CLAL4         VILQGSDCIETVAGASTERVSGVTFEYRLHSDSFSLYGSG-------DRRRGGKHHHSRE 376
O74743|IF4E2_SCHPO          ---------------------KETIEYSAHEDSSKAGSMRAK-----TR----------- 240
A0A099P4P5|A0A099P4P5_PICKU KRLAAKEG-----KNKVKPLAPMLFKYCTHQQDFLGDDKQPKKTCK-TVEYGEYDLVIEE 349
                                                    :.:    * .

P07260|IF4E_YEAST           ITL---------------------------------------- 213
Q6BHB4|Q6BHB4_DEBHA         --YNRRFTKPYHSHNHHNQNHSKNDSDN--------------- 442
A0A1D8PNX8|A0A1D8PNX8_CANAL QTYGNHYNRRYNKYNSNNSNNSNSNTASKTDSIAK------- 431
C4Y0X7|C4Y0X7_CLAL4         -------KEEQEK--DDQDNDDENNSAEKNGDISM------- 402
O74743|IF4E2_SCHPO          MSL---------------------------------------- 243
A0A099P4P5|A0A099P4P5_PICKU ITCNAQKSRRNRRR--QASNKA--FEGAESEDVNIIDRDAKV 387
```

(B)

Figgure S1. (**A**) Sequence alignment of eIF4E1 from different Saccharomycotina families and *Schizosaccharomyces pombe* (subfamily Taphrinomycotina).Labelled in red are the conserved tryptohane residues W1-W8, in yellow the conserved motif S/TVxxF next to W4, in blue *S. cerevisiae* S28 or equivalent serine phosphorylation sites of other yeast eIF4E1s. Examples for families Metschnikowiaceae or Debaryomycetaceae: *Clavispora lusitaniae* (CLAL4), *Candida albicans* (CANAL) or *Debaryomyces hansenii* (DEBHA); examples for family Pichiaceae: *Brettanomyces bruxellensis* (DEKBR), *Pichia kudriavzevii* (PICKU); examples for family Saccharomycetaceae: *Lachancea thermotolerans* (LACTC), *Saccharomyces cerevisiae* (YEAST). (**B**) Sequence alignment of eIF4E2 from different Saccharomycotina families and *Schizosaccharomyces pombe* of subphylum Taphrinomycotina as compared to *S. cerevisiae* eIF4E. Labelled in red are the conserved W1 to W8 or in grey the substitutions of W3, in yellow the conserved S/TVxxF

motif adjacent to W4 only found for *S. pombe* eIF4E2. Labelled in red is the conserved aminoterminal motif MSENLKRAESLFNRIMN found in different Saccharomycotina species. Examples for families Metschnikowiaceae or Debaryomycetaceae: *Clavispora lusitaniae* (CLAL4), *Candida albicans* (CANAL), *Debaryomyces hansenii* (DEBHA); examples for family Pichiaceae: *Brettanomyces bruxellensis* (DEKBR), *Pichia kudriavzevii* (PICKU) (corresponds to Figure 1A).

```
SP|P07260|IF4E_YEAST   ------------------------------------------------------------
TR|E9ADE1|E9ADE1_LEIMA ------------------------------------------------------------
TR|Q4QD60|Q4QD60_LEIMA ------------------------------------------------------------
TR|Q4Q813|Q4Q813_LEIMA MNPSAAAYIPQQSDAKGDP-----KSSSAAAVAKPPSTQPATKLSAAAEPFVPGGPKQMS 55
TR|Q4Q7R3|Q4Q7R3_LEIMA MNPNATEFMPGRRNGPDGGLEALPTSTADMELAKTPAGAA---AAAVHAPSLPGAVRRSL 57
TR|Q4Q217|Q4Q217_LEIMA ------------------------------------------------------------
TR|Q4Q9G7|Q4Q9G7_LEIMA ------------------------------------------------------------

SP|P07260|IF4E_YEAST   -----------------------------------------------------MSVEEVS 7
TR|E9ADE1|E9ADE1_LEIMA ------------------------------------------------------------
TR|Q4QD60|Q4QD60_LEIMA ------------------------------------------------------------
TR|Q4Q813|Q4Q813_LEIMA ATSTHVDPKATTEDEKTTAPLLMECPASSLPDSAAAAGAAKKEADENDDSQLDWLPEAQP 115
TR|Q4Q7R3|Q4Q7R3_LEIMA QNSPIIQPSRLSVKS------------------ASEIEAISKNSAL-NAAAAAYVPQRT- 97
TR|Q4Q217|Q4Q217_LEIMA ------------------------------------------------------------
TR|Q4Q9G7|Q4Q9G7_LEIMA ------------------------------------------------------------

SP|P07260|IF4E_YEAST   KKFEENVS--VDDTTATPKTVLSDSAHFDVKHPLNTKWTLWYTKPAVD-----KSE-SWS 59
TR|E9ADE1|E9ADE1_LEIMA --------------MSAPSSV--PPHKMANLHKLQRAWTLWYDSPSTY-----NTE-NWE 38
TR|Q4QD60|Q4QD60_LEIMA ----------MDPNTCAPASAVTDEQ---PLTLLWGTWEMWCDMPQRQQGQSTENT-NWL 46
TR|Q4Q813|Q4Q813_LEIMA TDWSESKL--PKLFGCHNT----AAKATSSAIPLHASWDLYADDHQGSSNMASNSSPTST 169
TR|Q4Q7R3|Q4Q7R3_LEIMA --LARVVLTQPSPLALAPS---EDPAKNNIEMMLDDLWCLFYLPTTLGENI-------KE 145
TR|Q4Q217|Q4Q217_LEIMA ----------------------MKSATMSATHALRDKWFVSFLPLLTADMVNTDYKGNWQ 38
TR|Q4Q9G7|Q4Q9G7_LEIMA ---------------MADSNPTKEGTSSAPLHPLKDKWFVFYIPASKGNEY--------- 36
                                                        *   * :

SP|P07260|IF4E_YEAST   DL--L--RPVTSFQTVEEFWAIIQNIPEPHELPLKSDYHVFRNDV-RPEWEDEANAKGGK 114
TR|E9ADE1|E9ADE1_LEIMA MS--L--VPIMTVHSVEEFFVMLRYMKPLHALRTSSQYHFFQEGV-KPMWEDPANKKGGK 93
TR|Q4QD60|Q4QD60_LEIMA EQ--V--KSIGLFDSAEGFWGIFNCTILPSQLPPNGSYYLFRKHI-APMWEHEANRRGGK 101
TR|Q4Q813|Q4Q813_LEIMA MS--FEPIFVSNVGDVESFWRLWRYLPAPSALPTVYTYSWFRKDI-KPEWEHPRNKKGGT 226
TR|Q4Q7R3|Q4Q7R3_LEIMA ED--YNPTLVFRVDSILTFWRVVNNIAAPSELQLS-TLYLFRDGI-DPKWEDPANRDGGI 201
TR|Q4Q217|Q4Q217_LEIMA LAAQERTQKLDWITSVEELWSTMNSLPKVHQLGMGSTLIFARNNKEPPSYEA--YPNGSR 96
TR|Q4Q9G7|Q4Q9G7_LEIMA ---EHETKELGYVSTIEEVYSTINTLPPITLLPNDDNLVFSRNKI-EPQFES--FPGGMR 90
                                :  .     .:   .       *         :.    * :*      *

SP|P07260|IF4E_YEAST   WSFQLRG------------------------KGADIDELWLRTLLAVIGETIDEDD---- 146
TR|E9ADE1|E9ADE1_LEIMA LWVNLDITSANGRSSNNNTSGTSAADGSAAEAKTDLDKAWENVLMATVGEYLDCVDKKDT 153
TR|Q4QD60|Q4QD60_LEIMA WVIPFTGKASRSE-----------------GDLQPVDEAWQTLCLSAIGELFPGDEE--- 141
TR|Q4Q813|Q4Q813_LEIMA ISIVVFDRDRSG-----------------LSDKQVLDDVFMAMLVGAVGESFHECST--- 266
TR|Q4Q7R3|Q4Q7R3_LEIMA VKVKA--------------------------TAAQVDEAWELLLCRTIGDSWSPSVR--- 232
TR|Q4Q217|Q4Q217_LEIMA IMINL-------------------------LKPPTTDAGLELVLAVVMGETAAEKASDG- 130
TR|Q4Q9G7|Q4Q9G7_LEIMA FSIFC-------------------------KTKTQCREALTYVVAVVLGEAIGRDACKG- 124
                         .                                           .:*:

SP|P07260|IF4E_YEAST   ---SQINGVVLSIRK-------------GGNKFALWTKSEDKEPLLRI-GGKFKQVLKLT 189
TR|E9ADE1|E9ADE1_LEIMA PTEPFVTGIVMSKRK-------------YHNRLAVWVSDASATDKIEALKKALTKEASLA 200
TR|Q4QD60|Q4QD60_LEIMA ----EICGVTVSRGRQRTLPSGHATSALSEWKLCLWTRSADNRGSQIRIAEYIRKQLHLQ 197
TR|Q4Q813|Q4Q813_LEIMA ----TLNGIMLKVRSNKP------------VTLQLWTAHSEV-GKLKAFANSVRDTLTKI 309
TR|Q4Q7R3|Q4Q7R3_LEIMA ---ETVNGVVLKVRER-------------AYWLELWVTKNSS-ALQKDLAEL----WHPI 271
TR|Q4Q217|Q4Q217_LEIMA ---KPVCDVLRIAARPSR-------EHSEQIRVEVWLSDSTRS---HAVAEFLAEAMRAK 177
TR|Q4Q9G7|Q4Q9G7_LEIMA ---ECVCDIVRIGHKGSA-------MYKESVRIEVWAHQSPYN---TAMEKYLVSTLSAI 171
                            : .:                       . :*

SP|P07260|IF4E_YEAST   ----DDGHLE-------------------------------------------------- 195
TR|E9ADE1|E9ADE1_LEIMA ----PIASMV-------------------------------------------------- 206
TR|Q4QD60|Q4QD60_LEIMA ----PPSKEAS--RDG------KSGEQDTLMEMPRSPDRSPVAKMREASGIPSAMTYVAH 245
TR|Q4Q813|Q4Q813_LEIMA MGAKTLQKLEYYSHHQ------KQAA-----------TNSLAARMKGKTKISPDHT---- 348
TR|Q4Q7R3|Q4Q7R3_LEIMA LGASFATT--YLTHAM------MQERSHA--------AAALAAEKQ-------------- 301
TR|Q4Q217|Q4Q217_LEIMA -----GLAANSYNIAEASFDAAAPGKDKKVLAASTMPSPSS----------P-P------ 215
TR|Q4Q9G7|Q4Q9G7_LEIMA ----PGITV-----SARPFK---------------------------------------- 182

SP|P07260|IF4E_YEAST   ---------FFPHSSANGRHPQPSITL--------- 213
TR|E9ADE1|E9ADE1_LEIMA ---------FTKHGEAS------------------- 214
TR|Q4QD60|Q4QD60_LEIMA RDLMEAKQEFVKGGSSVAQAFRPKYTLAIDVRNEGV 281
TR|Q4Q813|Q4Q813_LEIMA ---------F-------------------------- 349
TR|Q4Q7R3|Q4Q7R3_LEIMA ------------------KKNRRRY----------- 308
TR|Q4Q217|Q4Q217_LEIMA ---------MVKD----------------------- 219
TR|Q4Q9G7|Q4Q9G7_LEIMA ------------------------------------
```

Figure S2. Sequence alignment of *Leishmania major* eIF4E1 to eIF4E6 compared to *S. cerevisiae* eIF4E. Conserved W1 to W8 are labelled in red and substitutions of W2, W3, W4, W5, W6 and W7 are labelled in grey; highlighted is the conserved STVxxF motif adjacent to W4 (in yellow) found for *Leishmania major* eIF4E1 (corresponds to Figure 4B).

```
SP|P07260|IF4E_YEAST           MSVEEVSKKFEENVS--VDDTTATPKTVLSDSAHFDVKHPLNTKWTLWYT-KPAVDK-SE 56
TR|A0A0X8CYJ5|A0A0X8CYJ5_AMPCA MAVE-IGNPLPDGLGDTSERTDDADEGDVKDSTSLAPKHELQHHWCLWVHQRPGTQK-DG 58
TR|A0A109QBD6|A0A109QBD6_AMPCA ------------------------MAHRVNAGEFDPDEAPLETAWTFWSDKKTSDKKESD 36
TR|A0A0X9W7I2|A0A0X9W7I2_AMPCA MACLSFNENFNSNLKFMTDNSEE-------NDEMLKKDLPLRYNWSIWEQIMQSSDKANA 53
                                                              .     .  *.  * :*     . .* .

SP|P07260|IF4E_YEAST           SWSDLLRPVTSFQTVEEFWAIIQNIPEPHELPLKS--------------DYHVFRNDVRP 102
TR|A0A0X8CYJ5|A0A0X8CYJ5_AMPCA AWGDTQRMVHEFGTVEDFWCMFHYSYPPSKL-EHV--------------DYSLFKKGVTP 103
TR|A0A109QBD6|A0A109QBD6_AMPCA QYMEGLKQLGSFNTVQGFFRHYAWLQRATDLPRDH--------------SVLLFRKGYKP 82
TR|A0A0X9W7I2|A0A0X9W7I2_AMPCA QYSDATHKVASFSTVQEFWKLWNHMPQPSELLEQKRMVREQPDGLHVIDAIMIFRENIRP 113
                                : :  : : .* **: *:          .*  .                  :*::.  *

SP|P07260|IF4E_YEAST           EWEDEANAKGGKWSFQLRG--KGADIDELWLRTLLAVIGETI-DEDDSQINGVVLSIR-- 157
TR|A0A0X8CYJ5|A0A0X8CYJ5_AMPCA AWEDPAFKGGGRWVIKLEK-VKAQSLDDLWLSLCLALIGEAFIDIGGTLVCGAIVSVR-- 160
TR|A0A109QBD6|A0A109QBD6_AMPCA MWEE--FPDGGCWIVRIKRKLSPGFVNHMWENLMLACIGEAFAMPD---VVGCVLSTR-- 135
TR|A0A0X9W7I2|A0A0X9W7I2_AMPCA EWEDKWNATGGHFQFQLKPTVGGGQVDEYWNNLVLGMIGATIEPAG--MITGVRLVDKLS 171
                                **:     ** : .::.       ::. *    *. ** ::   .   : *  :  :

SP|P07260|IF4E_YEAST           ----KGGNKFALWTKSED-KEPLLRIGGKFKQVLKLTDDG------HLEF---FPH--SS 201
TR|A0A0X8CYJ5|A0A0X8CYJ5_AMPCA ----SRASKIALWLSQAKDEKKVMAIGREYRNVLASTPCLSDLATKELTF-EDFKK--QA 213
TR|A0A109QBD6|A0A109QBD6_AMPCA ----VKDDVLSIWNLSNRMGDTRFRIGEKLKEILALDMNALIQYKDHMQSLQDYSTYRNA 191
TR|A0A0X9W7I2|A0A0X9W7I2_AMPCA GPRAANVIRIEVWFSNYDDTQAVNTLKKNIEKCMATHLDGSVGTA---------PK---- 218
                                        : :*  .    .    :  : .: :

SP|P07260|IF4E_YEAST           ANGR--HPQPSITL---------------------------------------------- 213
TR|A0A0X8CYJ5|A0A0X8CYJ5_AMPCA VTFVLSRPQTQEATA----GVFQ------------------------------------- 232
TR|A0A109QBD6|A0A109QBD6_AMPCA KNYM-FAPSPSVTPQQHATPIMNQPADPSMDQDFDGLLPPAAIDDLLPPAAIDSEGFAAR 250
TR|A0A0X9W7I2|A0A0X9W7I2_AMPCA -NDTKAHSQPGGSKA----H----------------------------------------- 233
                                .      .   :

SP|P07260|IF4E_YEAST           -----
TR|A0A0X8CYJ5|A0A0X8CYJ5_AMPCA -----
TR|A0A109QBD6|A0A109QBD6_AMPCA GKFPA 255
TR|A0A0X9W7I2|A0A0X9W7I2_AMPCA -----
```

Figure S3. Sequence alignment of dinoflagellate eIF4Es from species *Amphidinium carterae* compared to *S. cerevisiae* eIF4E. In this alignment, the order of dinoflagellate eIF4E1a (accession number: A0A0X9W7I2_AMPCA) and eIF4E3a (accession number: A0A0X8CYJ5_AMPCA) is inverted. Conserved W1 to W8 are labelled (in red) and substitutions of W3, W4, and W6 are labelled (in grey); labelled is also the conserved S/TVxxF motif next to W4 found for dinoflagellate eIF4E1a, eIF4E2a and eIF4E3a (in yellow) (corresponds to Figure 5).
